# Peripheral Administration of IL-13 Induces Anti-inflammatory Microglial/Macrophage Responses and Provides Neuroprotection in Ischemic Stroke

**DOI:** 10.1007/s13311-019-00761-0

**Published:** 2019-08-01

**Authors:** Natalia Kolosowska, Meike H. Keuters, Sara Wojciechowski, Velta Keksa-Goldsteine, Mika Laine, Tarja Malm, Gundars Goldsteins, Jari Koistinaho, Hiramani Dhungana

**Affiliations:** 1 A.I.Virtanen Institute for Molecular Sciences, University of Eastern Finland, Kuopio, Finland; 2 Neuroscience Center, Helsinki Institute of Life Science (HiLIFE), University of Helsinki, Helsinki, Finland; 3 Neuroscience Center, HiLIFE, University of Helsinki, Haartmaninkatu 8, Helsinki, 00290 Finland

**Keywords:** Interleukin-13, stroke, neuroinflammation, microglia/macrophage polarization, functional recovery

## Abstract

Neuroinflammation is strongly induced by cerebral ischemia. The early phase after the onset of ischemic stroke is characterized by acute neuronal injury, microglial activation, and subsequent infiltration of blood-derived inflammatory cells, including macrophages. Therefore, modulation of the microglial/macrophage responses has increasingly gained interest as a potential therapeutic approach for the ischemic stroke. In our study, we investigated the effects of peripherally administered interleukin 13 (IL-13) in a mouse model of permanent middle cerebral artery occlusion (pMCAo). Systemic administration of IL-13 immediately after the ischemic insult significantly reduced the lesion volume, alleviated the infiltration of CD45^+^ leukocytes, and promoted the microglia/macrophage alternative activation within the ischemic region, as determined by arginase 1 (Arg1) immunoreactivity at 3 days post-ischemia (dpi). Moreover, IL-13 enhanced the expression of M2a alternative activation markers Arg1 and Ym1 in the peri-ischemic (PI) area, as well as increased plasma IL-6 and IL-10 levels at 3 dpi. Furthermore, IL-13 treatment ameliorated gait disturbances at day 7 and 14 and sensorimotor deficits at day 14 post-ischemia, as analyzed by the CatWalk gait analysis system and adhesive removal test, respectively. Finally, IL-13 treatment decreased neuronal cell death in a coculture model of neuroinflammation with RAW 264.7 macrophages. Taken together, delivery of IL-13 enhances microglial/macrophage anti-inflammatory responses *in vivo* and *in vitro*, decreases ischemia-induced brain cell death, and improves sensory and motor functions in the pMCAo mouse model of cerebral ischemia.

**Electronic supplementary material:**

The online version of this article (10.1007/s13311-019-00761-0) contains supplementary material, which is available to authorized users.

## Introduction

Despite intensive research during the last decades, ischemic stroke remains a severely dementing and disabling disease with limited options of effective therapy and stands among the major causes of death worldwide. The interruption of cerebral blood flow prompts a cessation of oxygen and nutrients supply, resulting almost instantly in excitotoxicity, oxidative stress, subsequent cellular damage, and apoptosis [1]. Upon the ischemic injury, the primary neuroinflammatory response is initiated by immune cells, such as rapidly activated resident microglia and macrophages, resulting in the local production of inflammatory cytokines and chemokines. In addition, secondary neuroinflammatory processes, widely initiated by polarized microglia and macrophages, astrocytes, and infiltrating peripheral leukocytes, can further aggravate the injury in the post-ischemic brain tissue [2]. During the early phase of stroke, activated microglia and infiltrated blood-derived macrophages secrete both pro- and anti-inflammatory factors, e.g., IL-1β, IL-6, tumor necrosis factor α (TNF-α), transforming growth factor beta (TGF-β), and activate pro-inflammatory enzymes, such as cyclooxygenase-2 (COX-2), and inducible nitric oxide synthase (NOS2/iNOS) [2–4]. Conventionally, macrophages and microglia are divided into the classical, pro-inflammatory M1 phenotype and the beneficial, anti-inflammatory M2 phenotype. Based on the distinct functions of microglia/macrophages and their gene expression profiles, M2 alternative activation state can be further classified into 4 subsets: M2a, M2b, M2c, and M2d [5]. M2a is typically induced by IL-4 or IL-13 and results in upregulation of arginase 1 (Arg1), chitinase-like 3 (Chil3/Ym1), found in inflammatory zone 1 (Fizz1), major histocompatibility complex II (MHC II) molecules, transforming growth factor β (TGF-β), IL-1 receptor antagonist (IL-1RA), and enhanced expression of scavenger receptors, e.g., mannose receptor (CD206) and scavenger receptor-A (CD204) [6]. The M2a profile has been associated with beneficial processes, such as neuroprotection, extracellular matrix remodeling and tissue repair, and increased phagocytic clearance of debris.

The patterns of microglia and macrophage activation vary substantially between the infarct core and the peri-ischemic (PI) zone. In the core area, microglia/macrophages acquire an anti-inflammatory phenotype during the early phase after stroke, but a large number of these cells undergoes apoptosis and dies within the next days. In contrast, the pro-inflammatory microglia and infiltrating macrophages are abundant in the PI area in the acute phase after stroke [7, 8]. 3 to 7 days post-ischemia (dpi), the anti-inflammatory microglial cells are predominant in the inflamed brain to promote tissue repair before they are gradually replaced by pro-inflammatory, M1-like polarized microglia/macrophages [7, 9, 10]. The number of M1-type microglia/macrophages in the PI area peaks around 2 weeks following stroke [10]. Therefore, the idea to change the fate of microglia and macrophages by shifting their activation state from M1 to M2 in the penumbra during an early phase of ischemic response may serve as a promising treatment strategy for ischemic stroke.

IL-13, an anti-inflammatory cytokine produced by Th2 cells, human naïve or memory CD4^+^ and CD8^+^ T cells, can inhibit the secretion of pro-inflammatory mediators, including nitric oxide (NO), IL-1β, IL-6, IL-12, and TNF-α [11], while enhancing the expression of mannose receptor and MHC II molecules [12]. In addition, IL-13 has been shown to suppress the infiltration of inflammatory cells and axonal loss [13]. Because IL-13 can effectively trigger a shift from M1 to M2a state [11, 14] and attenuate the production of inflammatory mediators [11], we aimed to elucidate whether IL-13 is able to reduce neuroinflammation and thus protect from ischemia-induced brain damage. Based on the previous data demonstrating IL-13’s beneficial, anti-inflammatory actions in some models of neurological disorders [15–17], testing the immunomodulatory potential of exogenous IL-13 administration was highly justified in the context of ischemic stroke. To demonstrate the beneficial effects of IL-13 in the cerebral ischemia, we employed a mouse model of permanent middle cerebral artery occlusion (pMCAo) with subsequent administration of murine recombinant IL-13. Intravenously administered, IL-13 changed the activation state of microglia/macrophages towards the M2a polarized, protective phenotype and provided functional recovery. Thus, our data show that IL-13 is neuroprotective and serves as a candidate for the treatment of ischemic stroke.

## Materials and Methods

### Animals

The National Animal Experiment Board of Finland approved all animal experiments, which were in accordance with the Council of Europe Legislation and Regulation for Animal Protection. A total of 77, 4-month-old male BALB/cOlaHsd mice (Envigo) were used in 4 independent experiments. The mice were housed under a 12-h light/dark cycle, with access to food and water ad libitum. All animals underwent a pMCAo surgery. The animals were randomized and divided into 2 groups of either IL-13 or vehicle phosphate buffered saline (PBS) treatment, using the GraphPad QuickCalcs software (GraphPad Software, Inc. La Jolla, CA, USA). All tests and data analyses were carried out blinded to the study groups.

### Experimental Setup

Mice for 3 independent studies were treated intravenously (i.v.) with 1 μg mouse recombinant IL-13 protein (ThermoFisher Scientific, Waltham, MA, USA) or vehicle (PBS) for immunohistochemical analyses at 3 dpi (vehicle/IL-13; *N* = 9), quantitative real-time PCR (qPCR) analyses at 3 dpi (vehicle/IL-13; *N* = 9), and behavioral testing (vehicle/IL-13; *N* = 9). Another set of animals was either treated with vehicle (*N* = 7), 2 μg (*N* = 8), or 5 μg (*N* = 8), to test the efficacy of slightly higher doses (study overview in Table 1). Altogether, 10 animals from vehicle-treated (*N* = 6) and IL-13-treated (*N* = 4) groups were excluded from further analyses due to bleeding during the surgery or otherwise inappropriate middle cerebral artery (MCA) occlusion.

**Table 1:** The number of animals for each experimental setup and the number of animals excluded due to an unsuccessful ischemia induction

	Total number of mice (*N*)	IHC	qPCR	Behavioral testing	Dosing scheme
Vehicle	34	9	9	9	7
IL-13	43				
1 μg	27	9	9	9	
2 μg	8				8
5 μg	8				8
Excluded vehicle	6	2	3	1	0
Excluded IL-13	4	1	2	1	0

### Ischemia Surgery and Treatment

All animals were initially anesthetized with 5% isoflurane and the surgical anesthesia was maintained with 2% isoflurane in 70% N_2_O/30% O_2_. The body temperature was maintained at 37 ± 0.5 °C with a homeothermic control system connected to a heating blanket and a rectal probe (Harvard apparatus; PanLab, Cornella, Spain). The left MCA was permanently occluded using a thermocoagulator (Aaron Medical Industries Inc., Clearwater, FL, USA) as described previously [18]. Briefly, a small skin incision was made between eye and ear and the temporal bone was exposed. A small burr hole of approximately 1 mm in diameter was drilled above the MCA and the dura was removed. Following the removal of the dura, the left MCA was lifted and cauterized, the muscle was repositioned, and the skin wound was sutured. After recovery from anesthesia, the animals received either PBS (in 100 μl volume) or IL-13 (1, 2, or 5 μg/animal; volume 100 μl) i.v. through the tail vein and were returned to normal cages. At 3 or 14 dpi, the mice were terminally anesthetized with 250 mg/kg of Avertin (Sigma-Aldrich, St. Louis, MO, USA) and perfused transcardially with 80 ml ice-cold saline containing 2500 IU/l heparin (LEO 5000 IU/mL, Leo Pharma A/S, Ballerup, Denmark).

### Magnetic Resonance Imaging

Magnetic resonance imaging (MRI) was performed at 3 dpi to image the brain infarct using a 9.4 T Oxford NMR 400 magnet (Oxford instrument PLC, Abington, UK). Multislice T2-weighted images (repetition time 3000 ms, echo time 40 ms, matrix size 128 × 256, and field of view 19.2 × 19.2 mm^2^, slice thickness 0.8 mm and number of slices 12) were obtained using a double spin-echo sequence with adiabatic refocusing pulse. The images were then analyzed using an in-house-made Aedes software under MATLAB environment (Math-works, Natick, MA, USA). The lesion volume was quantified using the formula: *Infarct volume = (volume of right hemisphere − (volume of left hemisphere − measured infarct volume)) / volume of right hemisphere.* The lesion volume is expressed as percentage [19].

### Immunohistochemistry (IHC)

The animals were sacrificed at 3 dpi by transcardial perfusion with ice-cold heparinized saline, and their brains were removed and post-fixed in 4% paraformaldehyde for 18–20 h, followed by cryopreservation in 30% sucrose for 48 h. The brains were frozen in liquid nitrogen and cut into 20 μm coronal sections using a cryostat (Leica Microsystems, Wetzlar, Germany). 6 consecutive sections spanning through the lesion, each 400 μm apart, were taken for analysis from each of the animals. Brain astrogliosis was visualized with glial fibrillary acid protein (GFAP) staining, microglia/macrophages were stained with ionized calcium-binding adapter molecule 1 (Iba1), M2-type microglia/macrophages were shown using arginase 1 (Arg1) staining, and leukocyte infiltration was demonstrated by leukocyte common antigen (CD45) staining. After blocking with 10% normal goat serum (Merck Millipore, Billerica, MA, USA), the brain sections were incubated with primary antibodies (GFAP, dilution 1:500, Dako, Glostrup, Denmark; Iba1, dilution 1:250, Wako Chemicals, Tokyo, Japan; Arg1, dilution 1:200, Santa Cruz Biotechnology, Dallas, TX, USA; CD45, dilution 1:100, Bio-Rad, Hercules, CA, USA; active cleaved caspase-3, dilution 1:200, Cell Signaling, Danvers, MA, USA) overnight at room temperature (RT). For Iba1 and Arg1 double staining, prior to primary antibody incubation, sections underwent antigen retrieval in aqueous 10 mM sodium citrate dihydrate solution preheated to 92 °C, pH 6. Following overnight incubation, the sections were washed with PBS containing 0.05% Tween 20 (Sigma-Aldrich) and incubated for 2 h at RT with secondary antibodies conjugated to Alexa Fluor 488 or 568 (dilution 1:200, ThermoFisher Scientific). Then slides were embedded with Vectashield mounting medium containing 4′,6-diamidino-2-phenylindole (DAPI). Sections for CD45 and caspase-3 antibody staining were incubated with biotinylated secondary antibody (dilution 1:200) for 2 h and thereafter with avidin-biotin complex reagent (all reagents from Vector Laboratories, Burlingame, CA, USA). Nickel-enhanced 3,3′-diaminobenzidine (DAB) was used for visualization of the immunoreactivity. For all IHC staining, negative controls were included in parallel sessions following the same procedures, except for the incubation with primary antibodies. To determine GFAP and Iba1 immunoreactivity, a region of interest (ROI) of 718 × 532 μm was imaged in the PI area at 10× magnification using AX70 microscope (Olympus Corporation, Tokyo, Japan) coupled to digital camera (Color View 12 or F-View; Soft Imaging System, Munster, Germany). For the Arg1 and cleaved caspase-3 staining, images of same size were taken from the ischemic core. For CD45 staining, images of equal size were taken from the corresponding border zone area, where majority of the immunoreactivity was observed. All immunoreactivites were quantified using ImagePro Plus Software (Media Cybernetics, Silver Spring, MD, USA). To quantify the immunoreactivity, a predefined intensity range was chosen to minimize the background noise or artifacts. We then calculated the percentage of immunoreactive areas. All analyses were done blinded to the study groups. The confocal images for Iba1/Arg1 colocalization analysis were acquired from a precisely defined PI area immediately adjacent to the ventral border of the ischemic lesion spanning across 3 consecutive sections (3, 4, and 5, starting at the rostral part of the infarct) under 20× magnification with Zeiss Axio Observer and Zeiss LSM 800 Airyscan module (Carl Zeiss AG, Jena, Germany). The representative whole coronal slice images and magnified images of immunoreactive areas from GFAP, Iba1/Arg1, and CD45 stainings were taken with 5× or 10× magnification, respectively on Zeiss Axio Imager 2 coupled to Axiocam digital camera (both Carl Zeiss AG) and using the Zen software. The representative confocal microphotographs illustrating Iba1/Arg1 colocalization were taken under 40× magnification with Zeiss Axio Observer and Zeiss LSM 800 Airyscan module (Carl Zeiss AG).

### Cytokine Secretion Analysis of Plasma

Buffered 129 mM sodium citrate was used as an anticoagulant in the volume ratio 1:9 of anticoagulant to blood. Collected blood samples were immediately centrifuged at 1500*g* for 15 min and plasma supernatants were additionally spun down at 13000*g* for 2 min to remove any trace of platelets. Plasma samples were aliquoted and stored at − 70 °C until analysis. To assess the cytokine concentration in plasma, Cytometric Bead Array (CBA) Mouse Inflammation Kit (BD Biosciences, San Jose, CA, USA) was used according to the manufacturer’s guidelines. The inflammation kit detects the following cytokines: IL-6, IL-10, monocyte chemoattractant protein-1 (MCP-1), IFN-γ, TNF, and IL-12p70. Data were acquired with FACSCalibur (BD Biosciences) or CytoFLEX S (Beckman Coulter, Indianapolis, IN, USA) and analyzed by the FCAP Array v2 software (Soft Flow Hungary Ltd., Pécs, Hungary).

### Quantitative Real-Time PCR Analyses of mRNA Levels

For quantification of gene expression, PI area, lesioned and contralateral cortex approximately corresponding to PI area were dissected at 3 dpi, snap-frozen in liquid nitrogen and stored at − 70 °C until analysis. Total RNA was isolated from brain tissue homogenates and primary murine microglia cultures with the mirVana miRNA Isolation Kit (ThermoFisher Scientific). Reverse transcription was performed with 500 ng of total RNA, Maxima reverse transcriptase, random hexamer primers, and dNTPs in the presence of ribonuclease inhibitor (all reagents ThermoFisher). The final cDNA concentration used for the gene expression analyses was 2.5 ng/μl. The relative expression levels of mRNAs encoding the selected genes were analyzed in duplicates and measured according to the manufacturer protocols by qPCR (StepOnePlus Real-Time PCR System, ThermoFisher Scientific) using the following specific TaqMan gene expression assays (ThermoFisher Scientific): Arg1 (Mm00479588_m1), Ym1 (Mm00657889_mH), Retnla (Mm00445109_m1), Il1b (Mm00434228_m1), Il6 (Mm00446190_m1), Il10 (Mm00439614_m1), Mertk (Mm00434920_m1), Lgals3 (Mm0080290_m1), Tnfa (Mm00443258_m1), Il18 (Mm00434225_m1), Ptgs2 (Mm00478374_m1), Ifng (Mm01168134_m1), and Marco (Mm00440265_m1). The results were normalized to the levels of endogenous control, eukaryotic 18S rRNA (ThermoFisher Scientific). Relative mRNA expression was calculated with the comparative 2^−∆∆Ct^ method, in which Ct is the threshold cycle number and results presented as fold changes in relation to the control conditions.

### Post-Surgery Assessment of the Locomotor Activity

#### Adhesive Removal Test

To assess general forepaw and mouth sensitivity (time-to-contact) and motor deficits (time-to-remove), we performed the adhesive removal test at 7 and 14 dpi as described previously [20]. 3 days prior to ischemia, mice were placed for 60 s in a 60 × 80-cm opaque plastic box for habituation. Next, a round adhesive sticker with 6.5 mm in diameter (Bel-Art Products, Wayne, NJ, USA) was placed on the hairless mid-paw area of each forelimb of the animal by applying equal pressure. The mouse was placed back into the opaque box and time was recorded. During one trial, the animal had 120 s time limit to remove the stickers. First sensing of the adhesive and the removal time were logged. After a short break, the animal was placed for a 30 s habituation into the box and adhesive tapes were placed on the forepaws in alternating fashion to the previous session. 3 trials per day and mouse were performed. Each mouse was trained once before baseline (BL) values were recorded. For the detection of possible somatosensory and motor deficits post-stroke, the median of time differences of sensing-to-removal between contra- and ipsilateral forepaws were calculated.

#### CatWalk Gait Analysis

The CatWalk automated gait analysis system (Noldus Information Technology, Wageningen, Netherlands) was used to assess gait and locomotor parameters post-ischemia. Mice were tested in a dimmed room (< 20 lx of illumination). For the assessment, an enclosed glass walkway (9 × 60 cm) was illuminated with a green, internally reflected light. 40 cm below the walkway, a high-speed camera captured the green light, reflected by the paws upon glass contact, and transformed it into a digital image [21]. The intensity threshold was set to 0.11, the camera gain was set to 18, and the maximum allowed speed variation was set to 50%. The mice walked spontaneously at their own speed and only uninterrupted runs were saved for analysis. The animals were trained twice before the BL recording on the day before pMCAo surgery. All mice underwent CatWalk testing at 7 and 14 dpi.

### Primary Microglia Cultures

Primary microglial cultures were prepared from C57BL/6J neonatal mice of 0–3 postnatal day as described elsewhere [22]. Briefly, mice were sacrificed by decapitation and their brains dissected. After that, tissue was mechanically dissociated and incubated in DMEM/F-12 supplemented with 1% penicillin/streptomycin and 0.05% Trypsin-EDTA (all ThermoFisher Scientific). Trypsin was inactivated with complete media DMEM/F-12 containing 10% heat-inactivated fetal bovine serum (iFBS), 1% penicillin/streptomycin (all ThermoFisher Scientific), the tissue homogenized, seeded on 15 cm dishes and left at 37 °C, 5% CO_2_ for 3 weeks. Thereafter, the astrocyte layer from mixed glial culture was trypsinized and the remaining microglia collected and plated on 48-well or 6-well plate format at the density of 125 × 10^3^ cells/well and 1 × 10^6^ cells/well, respectively. Microglia were treated with 20 ng/ml IFN-γ (Sigma-Aldrich) for 24 h followed by 10 ng/ml lipopolysaccharide (LPS, #L2630, serotype O111:B4, Sigma-Aldrich) for another 24 h (IFN-γ/LPS treatment referred as M1), in combination with or without 20 ng/ml IL-13 (ThermoFisher Scientific).

### MTT Viability and LDH Release Assays

The MTT reduction assay was performed 24 h after exposure to LPS as described earlier [23], with the following modifications. Briefly, following removal of the media, thiazolyl blue tetrazolium bromide (MTT, Sigma-Aldrich) was added to cells at a concentration of 1.2 mM, after which the cells were incubated for 3 h at 37 °C and 5% CO_2_, until visible purple crystals were formed. Thereafter, media were removed, cells were lysed, and formazan crystals were dissolved with DMSO (Fisher Scientific, Loughborough, UK). Absorbances were read at 585 nm with a Wallac Victor2 1420 microplate reader (Perkin Elmer, Waltham, MA, USA). The results were calculated as a percentage of relative MTT reduction compared to the control wells. The Pierce LDH Cytotoxicity Assay Kit was used according to the manufacturer’s instructions (ThermoFisher Scientific). Briefly, assay reaction mixture was added to media samples collected 24 h after LPS exposure. After 30 min incubation in the dark, stop solution was added to each sample and the absorbances were measured at 490 nm. The results are presented as percentage of LDH release in relation to LDH positive control.

### Flow Cytometry Analysis of N2a Cell Death in Coculture with RAW 264.7 Murine Macrophages

For coculture experiments, mouse neuroblastoma Neuro-2a (N2a) cells were seeded together with RAW 264.7 macrophages at a ratio of 1:1 and a density of 200 × 10^3^ cells/well on 12-well plate format, in DMEM supplemented with 10% iFBS and 1% P/S (all reagents ThermoFisher Scientific). 24 h after plating, cocultures were treated for 24 h with vehicle (PBS) or 25 ng/ml LPS (serotype O111:B4, Sigma-Aldrich) in combination with or without 20 ng/ml IL-13 (ThermoFisher Scientific). Next, the cocultures were exposed for another 24 h to vehicle or 100 ng/ml LPS and 25 ng/ml IFN-γ (Sigma-Aldrich) in combination with or without 20 ng/ml IL-13. Thereafter, the cells were collected and spun down and the media were saved for NO release measurements. Cells were incubated with CD11b-Alexa Fluor 647 antibody diluted 1:200 (BD Biosciences) for 30 min in the dark at 4 °C, then washed with HBSS containing 3% iFBS (ThermoFisher Scientific), resuspended in HBSS with 3% iFBS, and counterstained with propidium iodide at 2.5 μg/ml concentration (Sigma-Aldrich). To assess the percentage of dead N2a cells, the Alexa Fluor 647 and propidium iodide double staining was analyzed with CytoFLEX S (Beckman Coulter). The dead N2a cells were detected as Alexa Fluor 647 (CD11b) negative and propidium iodide positive (UL quadrant).

### Nitric Oxide Release Measurements

NO production was indirectly assessed as described previously [24] by detection of nitrites in media samples obtained from N2a and RAW 264.7 cocultures. A standard curve was prepared using 0–100 μM sodium nitrite (Sigma-Aldrich) in cell culture media. All samples in triplicates were transferred in 50 μl volume to a 96-well plate and 50 μl of Griess reagent was added per well. The absorbance was then measured with microplate reader Victor 2.0 (Perkin Elmer) at 544 nm and the nitrite concentration was calculated.

### Statistical Analyses

The statistical analyses were run with GraphPad Prism 5.03 (GraphPad Software, San Diego, CA, USA) using unpaired two-tailed *t* test or one-way ANOVA followed by the Bonferroni post hoc test to compare means of interest assuming homoscedasticity and normality of variables. Based on predetermined exclusion criteria, animals bleeding during the surgery or with otherwise inappropriate MCA occlusion were excluded from the study. Statistically significant outliers as calculated by Grubb’s test using the GraphPad Prism software were excluded from the datasets. Data are reported as mean ± SEM unless otherwise stated, the statistical test used and *N* numbers are stated in each figure legend. *P* values < 0.05 were considered statistically significant.

## Results

### IL-13 Treatment Significantly Decreased the Ischemic Lesion Volume

**Fig. 1:**
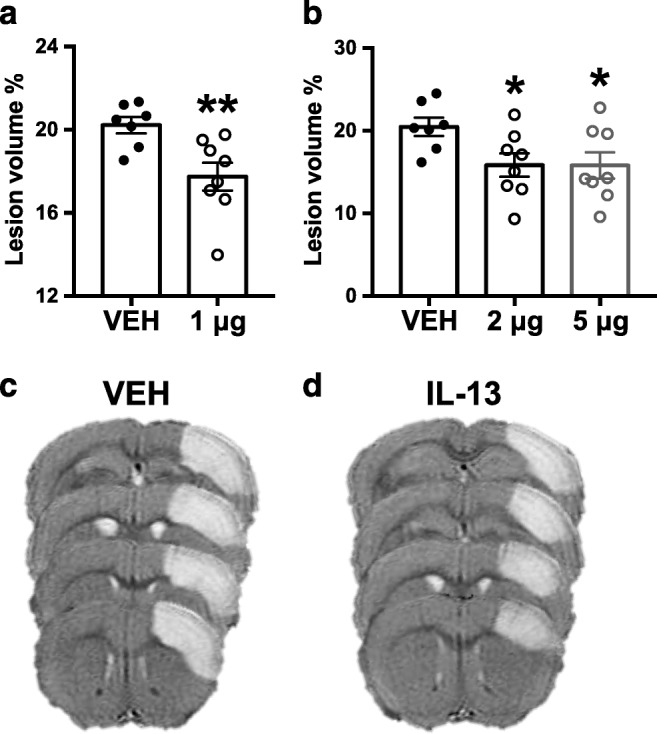
IL-13 treatment is protective against ischemia-induced cell loss. Infarct volumes were quantified from MRI images obtained at 3 dpi for vehicle-treated animals and animals treated with 1 μg of IL-13 (a). Lesion volumes were quantified from MRI images obtained at 3 days post-ischemia for vehicle-treated animals and animals treated with 2 or 5 μg of IL-13 (b). Representative MRI images of the infarcted brain in vehicle (c) and animals treated with 1 μg of IL-13 (d). Unpaired two-tailed *t* test (a) and one-way ANOVA followed by the Bonferroni post hoc test (b). Data are expressed as mean ± SEM. *p < 0.05, **p ˂ 0.01. VEH *N* = 7, IL-13 *N* = 8

To elucidate the neuroprotective role of IL-13 in ischemic stroke, mice received either 100 μl of PBS as vehicle or IL-13 at a dose of 1, 2, or 5 μg per animal right after cerebral ischemia induction. Based on MRI of the mouse brains at 3 dpi, we were able to determine the infarct size. Analysis of the infarct volumes revealed a significant reduction of the infarct volume at all tested IL-13 doses when compared to vehicle-treated mice, with 1 μg IL-13 dose decreasing the ischemic lesion size most significantly (Fig. 1a, *p* = 0.0091; Fig. 1b, *p* = 0.0327 and *p* = 0.0314).

### IL-13 Treatment Significantly Reduced Leukocyte Infiltration Without Altering Peri-ischemic Astrogliosis

**Fig. 2:**
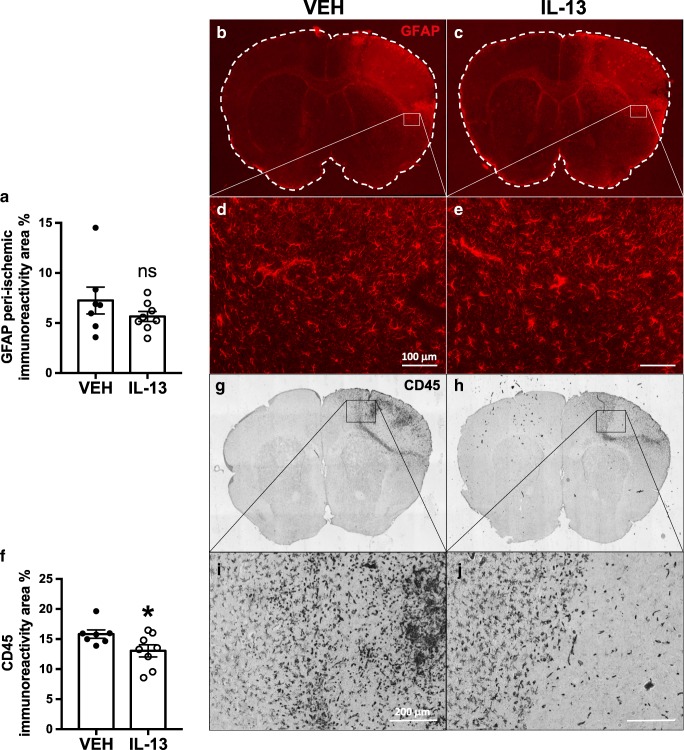
IL-13 administration did not induce any significant changes in astrogliosis, yet it significantly reduced the infiltration of CD45^+^ leukocytes in the ischemic brain parenchyma. Even though there was a trend towards reduced astrocytic activation, IL-13 did not alter GFAP expression significantly at 3 dpi (a). Representative images of entire coronal sections from vehicle-treated (b) and IL-13-treated (c) animals. Panels (d) and (e) represent GFAP immunoreactivity in the PI area of vehicle- and IL-13-treated mice, respectively. Scale bar 100 μm. IL-13 markedly alleviated CD45^+^ leukocytes infiltrating the ischemic lesion site (f). Representative images of entire coronal sections from vehicle-treated (g) and IL-13-treated (h) animals. Panel (i) shows a ROI in a representative brain slice of a vehicle-treated mouse, whereas (j) displays a corresponding ROI of an IL-13-treated mouse. Scale bar 200 μm. Unpaired two-tailed *t* tests. Data are expressed as mean ± SEM. *p ˂ 0.05. VEH *N* = 7, IL-13 *N* = 8

Analysis of GFAP immunoreactivity revealed that ischemic stroke upregulated the astrocyte activation significantly. This was seen in the PI area on the ipsilateral side of both treatment groups when compared to contralateral side (data not shown). However, IL-13 treatment failed to significantly alter the astrocytic activation and had only minor influence towards a decrease of GFAP expression (Fig. 2a, *p* = 0.2618).

Infiltrating leukocytes expressing high levels of CD45 are one of the hallmarks of inflammatory processes in the injured brain. Although highly expressed by monocytes/macrophages, CD45 is present to a lower extent in resting microglial cells [25]. In our analyses, a single injection of 1 μg IL-13 reduced significantly the number of CD45^+^ leukocytes infiltrated into the ischemic core at 3 days post-injury (Fig. 2f, *p* = 0.0461).

### IL-13 Treatment Increased the Type 2 Immune Responses of Microglia/Macrophages Within the Ischemic Brain

**Fig. 3:**
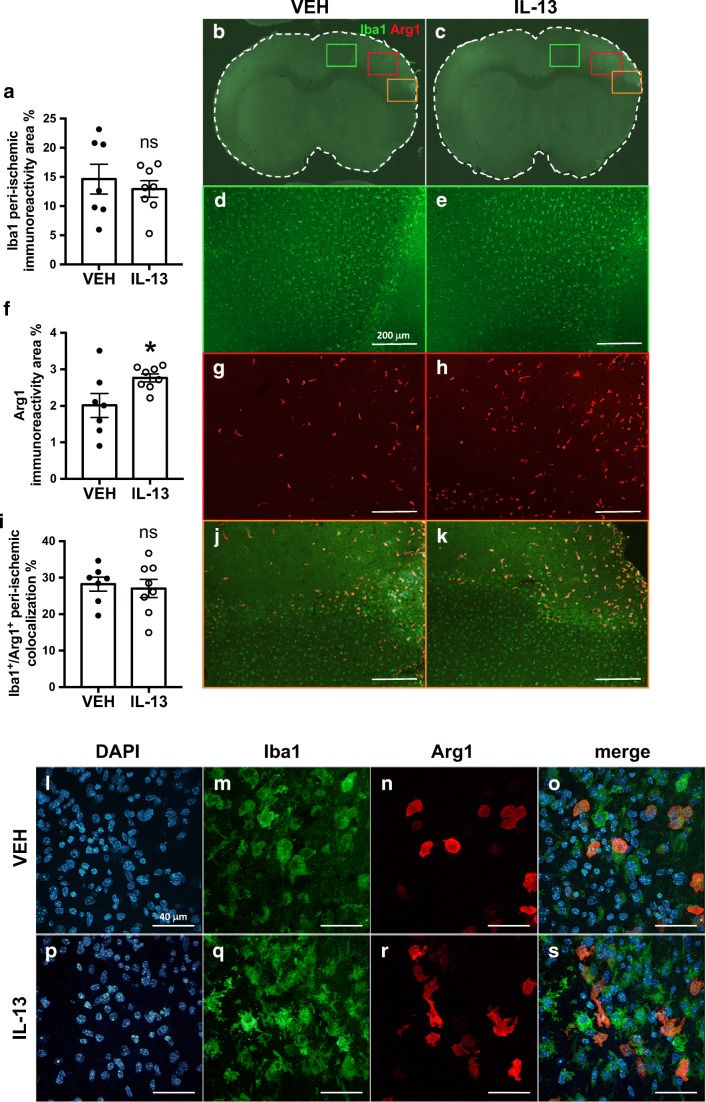
IL-13 did not change the expression of Iba1 in the peri-ischemic area, but induced Arg1 levels at 3 days post-stroke in the brains of mice subjected to permanent ischemia. IL-13 did not influence Iba1 expression in the PI area of the stroke brains (a). Representative images of entire coronal sections from vehicle-treated (b) and IL-13-treated (c) animals. Panels (d) and (e) represent the Iba1 immunoreactivity in the PI areas of vehicle- and IL-13-treated mice, respectively. Quantitative analysis revealed that IL-13 treatment significantly upregulated Arg1^+^ anti-inflammatory microglia/macrophages (f). The microphotographs represent the Arg1 immunoreactivity in the lesion areas of vehicle-treated (g) and IL-13-treated animals (h). IL-13 did not change the colocalization percentage of Iba1^+^ and Arg1^+^ microglia/macrophages in the PI area (i). Panels (j) and (k) depict Iba1^+^/Arg1^+^ colocalizing cells within the PI area adjacent to ischemic core of vehicle- and IL-13-treated mice, respectively. Scale bar 200 μm. Panels (l–s) consist of confocal microphotographs of the PI area from Iba1/Arg1 double stained brains of vehicle-treated (l–o) and IL-13-treated (p–s) stroke animals. Scale bar 40 μm. Unpaired two-tailed *t* tests. Data are expressed as mean ± SEM. *p ˂ 0.05. VEH *N* = 7, IL-13 *N* = 8

Microglia, the main resident immune cells in the brain, can acquire a pro-inflammatory phenotype (M1), but under specific conditions, including stimulation with IL-13, they have the ability to shift towards the alternative polarized phenotype (M2a). With Iba1 IHC staining we evaluated brain microglia/macrophages overall activation upon ischemic stroke. Although the Iba1 immunoreactivity was evidently increased after ischemic insult, we found no differences in Iba1 immunoreactivity between the vehicle and IL-13-treated mice at 3 dpi in the PI area (Fig. 3a, *p* = 0.5606). Using Arg1 IHC staining, we investigated whether the protective effect of IL-13 administration, observed in the PI and infarct area, is associated with a phenotypic shift of microglia/macrophages from M1 towards M2a. As shown in Fig. 3f–h, IL-13 treatment promoted a major increase of M2a phenotype, resembling microglia/macrophages following ischemic stroke in the ipsilateral hemisphere when compared to vehicle treatment (*p* = 0.0381). We also evaluated the colocalization percentage of Iba1^+^/Arg1^+^ cells in the PI area of the mouse brains and did not detect any differences between vehicle and IL-13-treated animals (Fig. 3i, l–s, *p* = 0.7203). The number of Iba1^+^ and Arg1^+^ cells specifically in the PI was also unchanged (data not shown). IL-13 treatment did not alter the immunoreactivity of cleaved caspase-3 in the PI area, indicating that this cytokine at 3 dpi is neither exerting its beneficial effect by preventing apoptosis nor further increasing the cell death in the ischemic region (data not shown).

### IL-13 Treatment Augmented the Expression of M2 Markers in the Ischemic Brain and Increased the Anti-inflammatory Cytokine Levels in the Plasma

**Fig. 4:**
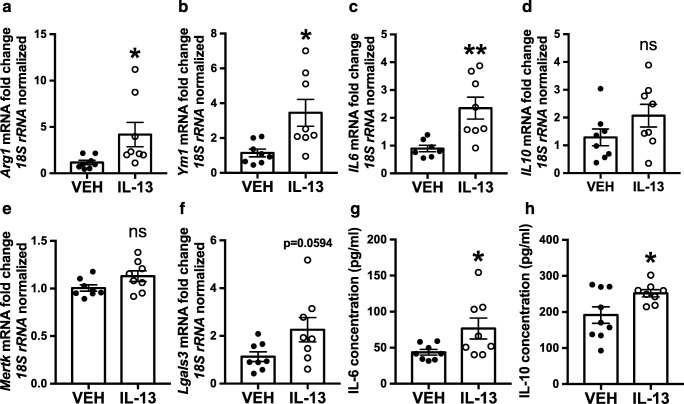
IL-13 administration *in vivo* induced the expression levels of myeloid alternative polarization markers both in the brain and in the periphery at 3 days post-stroke. qPCR analysis of the PI area of the ischemic brains of IL-13-treated mice revealed a marked increase of Arg1 (a), Ym1 (b), and IL-6 (c) transcripts. IL-13 did not have any significant effect on IL-10 (d), Mertk (e), and Lgals3 (f) expression levels in the PI brains of these mice. As evaluated by CBA, IL-6 (g) and IL-10 (h) cytokines were significantly elevated in the plasma by a single IL-13 injection post-stroke. Unpaired two-tailed *t* tests. Data are expressed as mean ± SEM. *p ˂ 0.05, **p ˂ 0.01. VEH *N* = 7–9, IL-13 *N* = 8

qPCR analysis of the PI area in the stroke brains at 3 dpi revealed a significant increase of major alternative activation markers Arg1 (Fig. 4a, *p* = 0.0408) and Ym1 (Fig. 4b, *p* = 0.0122), but not Fizz1/Rentla (data not shown) upon IL-13 treatment. IL-6 can act both as a pro-inflammatory and anti-inflammatory cytokine depending on its involvement with specific signaling pathways. It has been previously shown that classic IL-6 signaling via transmembrane IL-6R drives anti-inflammatory or regenerative processes [26]. As compared to the vehicle-treated stroke animals, IL-6 expression levels were markedly upregulated in the PI samples from the animals treated with IL-13 (Fig. 4c, *p* = 0.0055). IL-10 expression in the PI area of ischemic brains was not significantly affected by IL-13 administration (Fig. 4d, *p* = 0.1477). Phagocytosis-associated Mer receptor tyrosine kinase (Mertk) expression levels in PI were unaltered between IL-13 and vehicle animals (Fig. 4e, *p* = 0.0757); however, the upregulation of galectin-3 (Lgals3/ Gal3) transcript levels nearly reached significance in IL-13-treated mice (Fig. 4f, *p* = 0.0594). Gal3 deficiency has been reported to increase the neuronal apoptosis and lesion volume in ischemic stroke by impairing the microglial responses to the injury [4, 27]. In addition, we also checked whether IL-13 reduces gene expression levels of the major pro-inflammatory molecules including TNF-α, IFN-γ, Ptgs2/COX-2, IL-18, and macrophage receptor with collagenous structure (MARCO). However, we did not observe significant changes in expression of these molecules (data not shown). CBA analysis at 3 dpi showed that IL-13-treated mice had significantly upregulated IL-6 and IL-10 protein levels in their plasma (Fig. 4g, *p* = 0.0448 and 4 h, *p* = 0.0341). In contrast, the protein levels of pro-inflammatory cytokines including MCP-1, TNF-α, IFN-γ, and IL-12 were unaltered between IL-13- and vehicle-treated mice (data not shown).

### The Sensitivity of the Contralateral Forelimb Was Ameliorated After IL-13 Treatment in the Adhesive Removal Test

**Fig. 5:**
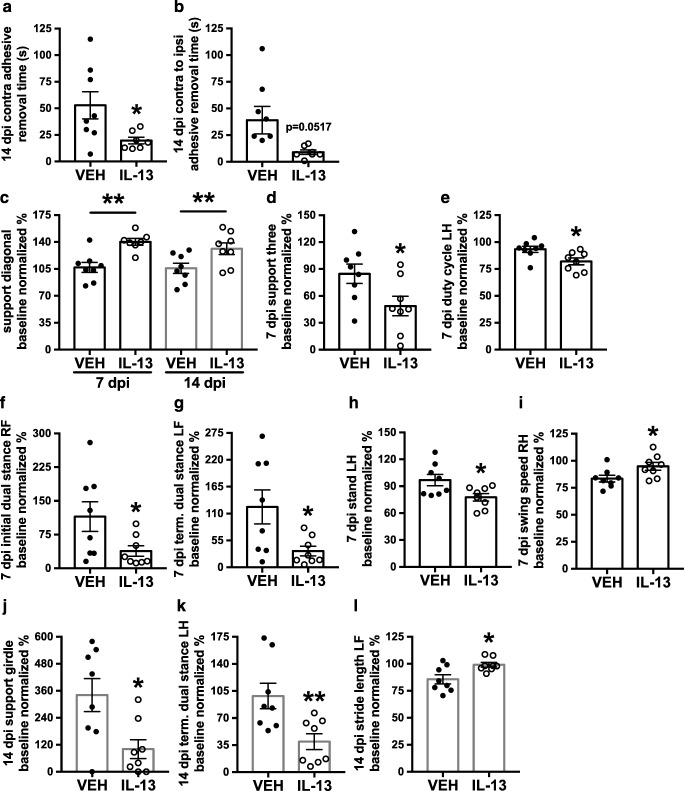
Behavioral improvement was observed after IL-13 treatment in the adhesive removal test and CatWalk gait analyses at subacute and late stages following ischemic stroke. The time of the adhesive tape removal from the contralateral forepaw was significantly reduced after IL-13 treatment when compared to untreated mice at 14 days post-ischemia (a). At the same time point after ischemic stroke, the IL-13-treated mice had shorter delay of removing the adhesive between contra- and ipsilateral side (b). In the CatWalk analyses, both at 7 and 14 dpi IL-13-treated mice displayed improved diagonal support (c). At 7 dpi, IL-13-treated mice showed decreased locomotor impairments as indicated by decreased values of support three (d) duty cycle of the left hind paw (e), initial dual stance of the right front paw (f), terminal dual stance of the left front paw (g), and the left hind paw’s stand parameters (h). Swing speed of the right hind paw (i) was substantially increased comparing to the control, vehicle-treated stroke group at 7 dpi. 14 days after ischemic insult, the mice from IL-13 group demonstrated markedly reduced support girdle (j) and terminal dual stance of the left hind limb (k), whereas their stride length of the left front paw was increased in comparison to the control group (l). Unpaired two-tailed *t* tests (a, b, d–l) and one-way ANOVA followed by the Bonferroni post hoc test (c). Data are expressed as mean ± SEM. *p ˂ 0.05, **p ˂ 0.01. VEH *N* = 8, IL-13 *N* = 8

The adhesive removal test was used to detect mouth and forepaw sensitivity and possible somatosensory deficits on the contralateral side, as well as motor performance of both mouth and forelimbs. Our results showed that IL-13 treatment significantly decreased the time needed to remove the adhesive from the contralateral front paw in the late stage after ischemic stroke, at 14 dpi (Fig. 5a, *p* = 0.0345). Furthermore, at the same time point post-stroke, there was a decrease in delay of removing the adhesive between contra- and ipsilateral side following IL-13 treatment, and this difference nearly reached statistical significance (Fig. 5b, *p* = 0.0517).

### Gait Analyses Revealed IL-13-Induced Improvements 7 and 14 Days After Ischemic Stroke

CatWalk automated gait analysis system was used to detect possible IL-13-induced motor improvements post-stroke. Both at 7 and 14 dpi, diagonal support, which is used the most in healthy conditions and accounts for 60–70% of the support types [28], was significantly ameliorated in the IL-13-treated group (Fig. 5c, *p* = 0.0015 and *p* = 0.0094, respectively). Some of the gait parameters of IL-13 animals were exclusively improved at 7 dpi. Support three, a parameter describing the number of paws used to support body weight during a step cycle, was reduced in the IL-13-treated group (Fig. 5d, *p* = 0.0349), as well as overall duty cycle (Fig. 5e, *p* = 0.0195), defined as Stand expressed as a percentage of step cycle [29]. Furthermore, initial dual stance of the right front (RF) paw (Fig. 5f, *p* = 0.0465), terminal dual stance of the left front (LF) paw (Fig. 5g, *p* = 0.0253), and stand of the left hind (LH) paw (Fig. 5h, *p* = 0.0242) were markedly shortened in IL-13-treated mice. Kinetic parameter swing speed of the right hind (RH) paw, meaning the velocity when paw is not in contact with the glass plate, was notably higher in the group of animals treated with IL-13 (Fig. 5i, *p* = 0.0336). 14 days after ischemic insult support girdle, an interlimb coordination parameter expressing the relative duration of contact with the glass plate of 2 paws simultaneously (Fig. 5j, *p* = 0.0129), as well as terminal dual stance of the ipsilateral hind paw (Fig. 5k, *p* = 0.0086) were reduced by IL-13 administration. Finally, at 14 dpi, the stride length of the ipsilateral front paw, defining the distance between successive placements of a paw, was significantly increased in the IL-13 group (Fig. 5l, *p* = 0.0125).

### IL-13 Treatment *In Vitro* Promoted the Alternative Activation of Primary Microglia Concomitantly Exposed to Pro-inflammatory Conditions

**Fig. 6:**
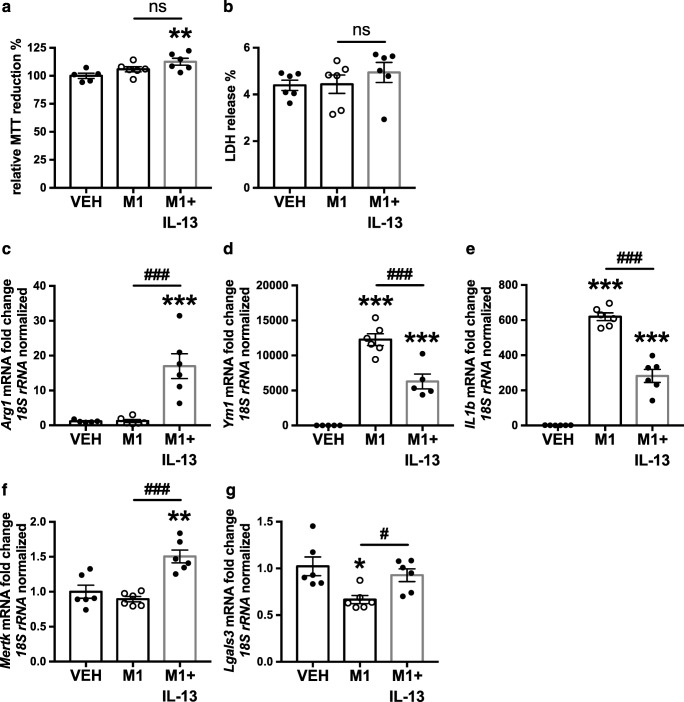
IL-13 promotes the shift of primary microglia polarization towards M2 phenotype. The primary microglia viability measured by their metabolic capacity of MTT reduction (a) and treatment cytotoxicity assessed by LDH release (b) remained unaltered between M1 and M1 + IL-13-treated cells. Concomitant IL-13 treatment with IFN-γ/LPS exposure (M1 + IL-13) caused a robust increase of Arg1 expression in the primary microglial culture as compared to M1 treatment alone (c). Although the same treatment paradigm caused a significant decrease of Ym1 transcript levels as compared to M1 exposure alone, Ym1 levels in M1 + IL-13 group remained upregulated in comparison to the vehicle-treated microglia (d). IL-13 exposure concomitant with M1 challenge was able to alleviate the pro-inflammatory IL-1β expression as compared to M1 microglia (e). The levels of phagocytosis-related Mertk (f) and Lgals3 (g) in M1 + IL-13 microglial cells were notably increased. Cytotoxicity assays were repeated 3 times. One-way ANOVA followed by Bonferroni post hoc tests. Data are expressed as mean ± SEM. *p ˂ 0.05, **p ˂ 0.01, ***p ˂ 0.001 *versus* vehicle-treated cells and ^###^*p* < 0.001 as compared to M1-treated cells. VEH *N* = 5–6, M1 *N* = 6, M1 + IL-13 *N* = 5–6

We first looked at the microglial viability under pro-inflammatory conditions and assessed the potential cytotoxicity of IFN-γ/LPS (M1)/IL-13 treatment. The microglial metabolic capacity of MTT reduction remained unaltered when compared between M1 and M1/IL-13 exposure (Fig. 6a, *p* = 0.0871), and the amount of released LDH was unchanged among the treatment types (Fig. 6b, *p* = 0.3374), indicating that IL-13 had no impact on cell viability under inflammation and was not toxic to the activated microglia.

qPCR analysis of the murine primary microglial cultures exposed to a combination of M1 and IL-13 revealed a substantial increase of M2-type marker Arg1 (Fig. 6c, *p* = 0.0001) and strong downregulation of pro-inflammatory IL-1β transcript levels (Fig. 6e, *p* < 0.0001), compared to M1 treatment alone. Ym1, considered as another marker of M2 activation, was significantly upregulated upon IFN-γ/LPS stimulation even without IL-13 treatment (Fig. 6d, *p* = 0.0001). IL-13 given together with M1 exposure upregulated MerTK (Fig. 6f, *p* < 0.0001) and Lgals3 (Fig. 6g, *p* = 0.0255) expression, molecules shown to interact and participate in microglial phagocytosis [30, 31].

### IL-13 Exposure *In Vitro* Protected Neuronal N2a Cells from Inflammation-Induced Death in Coculture with RAW 264.7 Macrophages

**Fig. 7:**
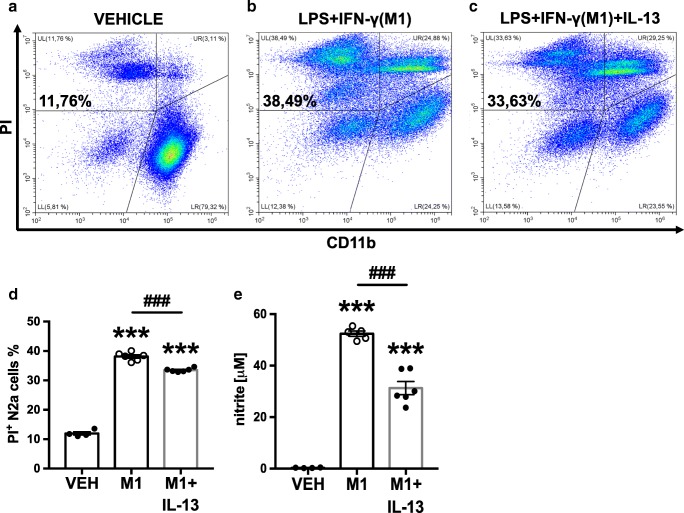
IL-13 decreases N2a neuroinflammation-induced cell death in a coculture model with RAW 264.7 macrophages. In comparison to vehicle-treated cells (a), the proportion of CD11b negative, propidium iodide positive (PI^+^), and dead N2a cells was increased by about 26% in LPS and IFN-γ exposed cocultures (b). IL-13 treatment combined with LPS and IFN-γ markedly decreased the percentage of PI^+^ N2a cells as compared to LPS and IFN-γ challenge alone (c, d). NO production in N2a and RAW 264.7 cocultures under pro-inflammatory conditions was notably attenuated by IL-13 exposure (e). One-way ANOVA followed by Bonferroni post hoc tests. Data are expressed as mean ± SEM. ***p ˂ 0.001 *versus* vehicle-treated cells and ^###^*p* < 0.001 as compared to M1-treated cells. VEH *N* = 4, M1 *N* = 6, M1 + IL-13 *N* = 6

To investigate the neuroprotective properties of IL-13 in an *in vitro* coculture model of N2a and RAW 264.7 macrophages, cocultures were exposed to vehicle, LPS, and IFN-γ (M1) and M1 combined with IL-13 (Fig. 7a–d). 48 h of M1 stimulation induced the N2a cell death by about 26% in comparison to vehicle-treated cells (*p* < 0.0001), whereas IL-13 treatment combined with M1 potently decreased the percentage of dead N2a cells compared to M1 treatment alone (*p* < 0.0001). Moreover, NO release in N2a and RAW 264.7 cocultures challenged with pro-inflammatory stimulation was significantly alleviated by IL-13 treatment (Fig. 7e, *p* < 0.0001).

## Discussion

Controlling neuroinflammation, especially the processes of inflammation-induced secondary brain damage, is crucial to improve the ischemic stroke outcome. Here, for the first time, we demonstrate that i.v. administration of exogenous recombinant murine IL-13 is beneficial in a mouse model of pMCAo. A single peripheral injection of IL-13 markedly reduced the ischemic brain damage, elicited anti-inflammatory responses in the injured brain, and provided long-term functional improvement by ameliorating sensorimotor deficits. IL-13 has been demonstrated to mediate a phenotypic shift from pro-inflammatory M1-type microglia/macrophages towards the anti-inflammatory M2-phenotype in a number of studies [14–16, 32–34]. Importantly, mesenchymal stem cell (MSC)-based IL-13 delivery orchestrated alternative polarization of macrophages and resulted in improved functional recovery following SCI [32]. Most recently, IL-13-overexpressing MSCs were found to enhance the transmigration of alternatively activated macrophages into the infarct area [17]. These data support our finding that IL-13 promotes the phenotypic shift of microglia/macrophages towards the anti-inflammatory type M2a, hence providing the neuroprotection and long-term functional recovery.

In this study, IL-13 treatment provided a significant neuroprotection by reducing the ischemic infarct size when administered right after the ischemic onset. Irrespective of the doses we used in our study, we found a consistent reduction in lesion volume. IL-13 treatment did not significantly change the overall Iba1 immunoreactivity of microglia/macrophages in the PI area at 3 dpi. Instead, we detected an increased Arg1 immunoreactivity within the infarct area in mice treated with IL-13, suggesting the importance of alternatively activated infiltrating macrophages in neuroprotection. In fact, cell-based delivery of IL-13 has been demonstrated to promote the alternative polarization of microglia and/or macrophages in the mouse model of SCI and tMCAo [17, 32]. Although we did not observe an increased percentage of the cells immunoreactive for both Iba1^+^ and Arg1^+^ in the PI area of the animals treated with IL-13, the Arg1 transcript levels in this area were notably increased, which is in line with the previous studies [34, 35]. In addition, our data showed a decrease of CD45^**+**^ infiltrating leukocytes in the infarcted brains of IL-13-treated mice. Thus, the protective effect of IL-13 in ischemic stroke could be partially explained by the reduced infiltration of peripheral immune cells and increased proportion of M2-skewed cells in the ipsilateral hemisphere. Astrogliosis was not substantially affected by IL-13 treatment, as we observed similar levels of GFAP immunoreactivity following ischemia in vehicle and IL-13-treated mice.

Peripheral inflammation is known to exacerbate the ischemic damage [18, 36]. As such, the modulation of peripheral inflammation has been shown to provide neuroprotection in a number of studies [35, 37–39]. Therefore, we analyzed whether IL-13 treatment modulates the peripheral immune response after brain ischemia. Indeed, we observed increased levels of the anti-inflammatory cytokine IL-10 in plasma. Although subject to debate, there are studies indicating that low levels of IL-10 in plasma correlate with neurological deterioration [40], which would support our findings. IL-6 can be considered either a pro- or anti-inflammatory cytokine, depending on its signaling pathway [26]. This cytokine was upregulated both on the mRNA level in the PI area and on the protein level in the plasma of IL-13-treated animals. The anti-inflammatory role and thus the protective effect of IL-6 in ischemic stroke is supported by a previous study, in which repetitive i.v. administration of IL-6 protein ameliorated the behavioral outcome after pMCAo in mice [41]. However, IL-13 was unable to alter the levels of major pro-inflammatory molecules like TNF-α and IFN-γ in both CNS and periphery. This can be explained partially by the fact that IL-13 does not inhibit the pro-inflammatory mediators but rather induces the expression of molecules associated with type 2 immune response [5].

Ischemic stroke is a highly debilitating disease and the functional recovery is warranted by the therapeutic treatments. IL-13 was able to ameliorate behavioral deficits at subacute and chronic stages compared to control mice as evident in CatWalk gait analyses and adhesive removal test. At 7 dpi, IL-13-treated mice showed notable improvements in several parameters associated with locomotor abilities as reported previously [29, 42]. The support diagonal, the type of limb support dominant in the healthy conditions [28], was improved at both 7 and 14 dpi. At 14 dpi, IL-13-treated mice no longer required to compensate abnormal movements of the contralateral hind limb and showed improved coordination patterns during the step cycle [29]. Finally, the adhesive removal test displays yet another improvement of the stroke-induced sensorimotor deficits in the IL-13-treated group.

In addition to modulation of the infiltrating peripheral immune cells, IL-13 is expected to cross the damaged BBB to some extent and thus act on CNS immune cells. In fact, both IL-4 and IL-13 have been shown to mediate the alternative microglial activation in the brain [43]. Therefore, we tested the effect of IL-13 in *in vitro* primary microglia following M1-type (IFN-γ combined with LPS) stimulation. As expected, microglial cells challenged with M1 stimulation responded with a robust upregulation of the M1-like activation marker IL-1β [44]. IL-13 treatment with simultaneous M1 stimulation alleviated IL-1β expression and concomitantly induced transcript levels of M2-associated marker Arg1, which is in agreement with our *in vivo* data. Intriguingly, Ym1 was significantly upregulated with M1 stimulation alone. In fact, a previous study has shown that LPS exposure increased the expression of Ym1 in rat primary microglia 24 h after stimulation, suggesting that Ym1 is not a typical M2 marker for these cells [45]. In addition, combined IL-13 and M1 stimulation increased MerTK and Gal3 levels, a receptor and its ligand known to be associated with the phagocytic activity of microglial cells [30, 31]. However, these results could not be obtained from the brains of IL-13-treated mice subjected to permanent ischemia, as the expression of MerTK remained unaffected in the PI regions, and the moderate increase in Gal3 levels failed to reach statistical significance. Likewise, microglial viability under inflammation was unaltered by IL-13, as evident by the results of MTT reduction and LDH release assays *in vitro* and caspase-3 immunoreactivity analysis in *in vivo* studies, which is in contrast to the previous findings reported by others [46, 47]. These discrepancies may be partially explained due to the differences in the models, IL-13 doses and the time points used.

Our *in vivo* data suggested that IL-13-mediated neuroprotection in ischemic stroke might be exerted by alternatively polarized peripheral immune cells infiltrating the ischemic infarct site. Based on this observation, we employed an *in vitro* neuroinflammation model using RAW 264.7 macrophages cocultured with N2a cells. The percentage of propidium iodide-positive (dead) N2a cells was markedly lower in cocultures exposed to IFN-γ, LPS, and IL-13 compared to cocultures challenged only with IFN-γ and LPS. NO is either directly neurotoxic or induces neuronal death through generated peroxynitrite, which inhibits mitochondrial respiration and elicits glutamate release resulting in excitotoxicity and neuronal apoptosis [48]. In our model, NO released from the cocultures was significantly attenuated by IL-13 under inflammatory condition. This might be attributed to the increased expression of Arg1 by macrophages, which subsequently reduces the bioavailability of arginase, the substrate for iNOS. Although we did not measure the concentration of cytokines from coculture media, the possible reduction of pro-inflammatory cytokine levels by IL-13 cannot be ruled out.

There is an increasing evidence that induction of Th2-type immune response has neuroprotective effects following stroke by limiting progression of the ischemic infarct [49–52]. However, repetitive administration of M2 cytokines after ischemic stroke can enhance the post-stroke immunosuppression, thereby facilitating bacterial infections and increasing mortality [53, 54]. In our study, we used a single dose of IL-13 in a pMCAo model that produces strictly cortical lesions. Hence, we expected a minor immunosuppression. Whether IL-13 treatment is efficient at a clinically relevant time point in more severe models of ischemia where acute post-stroke immunosuppression is observed remains unknown.

Our study reports for the first time in the animal model of ischemic stroke the beneficial actions of i.v. administered anti-inflammatory cytokine IL-13. Apart from decreasing the ischemic infarct size, the histological analyses revealed that a single dose of IL-13 administered immediately post-stroke markedly induced the proportion of M2-type microglia/macrophages in the ischemic area of the brain and significantly ameliorated the long-term sensorimotor deficits. Importantly, our findings have potential implications in the clinical practice and therefore IL-13 treatment can be considered a novel therapeutic approach for the treatment of cerebral ischemia.

## Electronic Supplementary Material

ESM 1 (PDF 1224 kb)

## References

[1] Liu R, Pan M, Tang J (2017). Role of neuroinflammation in ischemic stroke. Neuroimmunol Neuroinflammation..

[2] Dhungana H, Huuskonen MT, Jaronen M (2017). Sulfosuccinimidyl oleate sodium is neuroprotective and alleviates stroke-induced neuroinflammation. J Neuroinflammation..

[3] Schielke GP, Yang G-Y, Shivers BD, Betz AL (1998). Reduced ischemic brain injury in interleukin-1β converting enzyme-deficient mice. J Cereb Blood Flow Metab..

[4] Lalancette-Hébert M, Swarup V, Beaulieu JM (2012). Galectin-3 is required for resident microglia activation and proliferation in response to ischemic injury. J Neurosci..

[5] Mantovani A, Sica A, Sozzani S, Allavena P, Vecchi A, Locati M (2004). The chemokine system in diverse forms of macrophage activation and polarization. Trends Immunol..

[6] Rőszer T. Understanding the mysterious M2 macrophage through activation markers and effector mechanisms. Mediators Inflamm. 2015; 2015:816460. 10.1155/2015/816460 PMC4452191 26089604

[7] Taylor RA, Sansing LH (2013). Microglial responses after ischemic stroke and intracerebral hemorrhage. Clin Dev Immunol..

[8] Perego C, Fumagalli S, De Simoni MG (2011). Temporal pattern of expression and colocalization of microglia/macrophage phenotype markers following brain ischemic injury in mice. J Neuroinflammation..

[9] Schroeter M, Jander S, Witte OW, Stoll G (1999). Heterogeneity of the microglial response in photochemically induced focal ischemia of the rat cerebral cortex. Neuroscience..

[10] Hu X, Li P, Guo Y (2012). Microglia/macrophage polarization dynamics reveal novel mechanism of injury expansion after focal cerebral ischemia. Stroke..

[11] Doherty TM, Kastelein R, Menon S, Andrade S, Coffman RL (1993). Modulation of murine macrophage function by IL-13. J Immunol..

[12] Gordon S (2003). Alternative activation of macrophages. Nat Rev Immunol..

[13] Ochoa-Repáraz J, Rynda A, Ascón MA (2008). IL-13 production by regulatory T cells protects against experimental autoimmune encephalomyelitis independently of autoantigen. J Immunol..

[14] Cardilo-Reis L, Gruber S, Schreier SM (2012). Interleukin-13 protects from atherosclerosis and modulates plaque composition by skewing the macrophage phenotype. EMBO Mol Med..

[15] Le Blon D, Guglielmetti C, Hoornaert C (2016). Intracerebral transplantation of interleukin 13-producing mesenchymal stem cells limits microgliosis, oligodendrocyte loss and demyelination in the cuprizone mouse model. J Neuroinflammation..

[16] Guglielmetti C, Le Blon D, Santermans E (2016). Interleukin-13 immune gene therapy prevents CNS inflammation and demyelination via alternative activation of microglia and macrophages. Glia..

[17] Hamzei Taj S, Le Blon D, Hoornaert C (2018). Targeted intracerebral delivery of the anti-inflammatory cytokine IL13 promotes alternative activation of both microglia and macrophages after stroke. J Neuroinflammation..

[18] Dhungana H, Malm T, Denes A (2013). Aging aggravates ischemic stroke-induced brain damage in mice with chronic peripheral infection. Aging Cell..

[19] Shuaib A, Xu Wang C, Yang T, Noor R (2002). Effects of nonpeptide V(1) vasopressin receptor antagonist SR-49059 on infarction volume and recovery of function in a focal embolic stroke model. Stroke..

[20] Bouet V, Boulouard M, Toutain J (2009). The adhesive removal test: a sensitive method to assess sensorimotor deficits in mice. Nat Protoc..

[21] Parkkinen S, Ortega FJ, Kuptsova K, Huttunen J, Tarkka I, Jolkkonen J. Gait impairment in a rat model of focal cerebral ischemia. Stroke Res Treat. 2013; 2013:410972. 10.1155/2013/410972 PMC3603709 23533959

[22] Malm T, Mariani M, Donovan LJ, Neilson L, Landreth GE (2015). Activation of the nuclear receptor PPARδ is neuroprotective in a transgenic mouse model of Alzheimer’s disease through inhibition of inflammation. J Neuroinflammation..

[23] Denizot F, Lang R (1986). Rapid colorimetric assay for cell growth and survival. Modifications to the tetrazolium dye procedure giving improved sensitivity and reliability. J Immunol Methods..

[24] Green LC, Wagner DA, Glogowski J, Skipper PL, Wishnok JS, Tannenbaum SR (1982). Analysis of nitrate, nitrite, and [15N]nitrate in biological fluids. Anal Biochem..

[25] Ritzel RM, Patel AR, Grenier JM (2015). Functional differences between microglia and monocytes after ischemic stroke. J Neuroinflammation..

[26] Scheller J, Chalaris A, Schmidt-Arras D, Rose-John S (1813). The pro- and anti-inflammatory properties of the cytokine interleukin-6. Biochim Biophys Acta - Mol Cell Res..

[27] Lalancette-Hebert M, Gowing G, Simard A, Weng YC, Kriz J (2007). Selective ablation of proliferating microglial cells exacerbates ischemic injury in the brain. J Neurosci..

[28] Bernardes D, Oliveira ALR (2017). Comprehensive CatWalk gait analysis in a chronic model of multiple sclerosis subjected to treadmill exercise training. BMC Neurol..

[29] Caballero-Garrido E, Pena-Philippides JCC, Galochkina Z, Erhardt E, Roitbak T (2017). Characterization of long-term gait deficits in mouse dMCAO, using the CatWalk system. Behav Brain Res..

[30] Caberoy NB, Alvarado G, Bigcas J-L, Li W (2012). Galectin-3 is a new MerTK-specific eat-me signal. J Cell Physiol..

[31] Nomura K, Vilalta A, Allendorf DH, Hornik TC, Brown GC (2017). Activated microglia desialylate and phagocytose cells via neuraminidase, galectin-3, and Mer tyrosine kinase. J Immunol..

[32] Dooley D, Lemmens E, Vangansewinkel T (2016). Cell-based delivery of interleukin-13 directs alternative activation of macrophages resulting in improved functional outcome after spinal cord injury. Stem cell reports..

[33] Hofmann U, Knorr S, Vogel B (2014). Interleukin-13 deficiency aggravates healing and remodeling in male mice after experimental myocardial infarction. Circ Hear Fail..

[34] Offner H, Subramanian S, Wang C (2005). Treatment of passive experimental autoimmune encephalomyelitis in SJL mice with a recombinant TCR ligand induces IL-13 and prevents axonal injury. J Immunol..

[35] Rosenzweig HL, Lessov NS, Henshall DC, Minami M, Simon RP, Stenzel-Poore MP (2004). Endotoxin preconditioning prevents cellular inflammatory response during ischemic neuroprotection in mice. Stroke..

[36] Marcet P, Santos N, Borlongan CV (2017). When friend turns foe: central and peripheral neuroinflammation in central nervous system injury. Neuroimmunol Neuroinflammation..

[37] Pennypacker KR (2014). Targeting the peripheral inflammatory response to stroke: role of the spleen. Transl Stroke Res..

[38] Ahmed SH, He YY, Nassief A (2000). Effects of lipopolysaccharide priming on acute ischemic brain injury. Stroke..

[39] Seifert HA, Leonardo CC, Hall AA (2012). The spleen contributes to stroke induced neurodegeneration through interferon gamma signaling. Metab Brain Dis..

[40] Vila N, Castillo J, Dávalos A, Esteve A, Planas AM, Chamorro A (2003). Levels of anti-inflammatory cytokines and neurological worsening in acute ischemic stroke. Stroke..

[41] Grønhøj MH, Clausen BH, Fenger CD, Lambertsen KL, Finsen B (2017). Beneficial potential of intravenously administered IL-6 in improving outcome after murine experimental stroke. Brain Behav Immun..

[42] Encarnacion A, Horie N, Keren-Gill H, Bliss TM, Steinberg GK, Shamloo M (2011). Long-term behavioral assessment of function in an experimental model for ischemic stroke. J Neurosci Methods..

[43] Mori S, Maher P, Conti B. Neuroimmunology of the interleukins 13 and 4. Brain Sci. 2016; 6. 10.3390/brainsci6020018 PMC4931495 27304970

[44] Kaushik DK, Thounaojam MC, Kumawat KL, Gupta M, Basu A (2013). Interleukin-1β orchestrates underlying inflammatory responses in microglia via Krüppel-like factor 4. J Neurochem..

[45] Lively S, Schlichter LC (2018). Microglia responses to pro-inflammatory stimuli (LPS, IFNγ+TNFα) and reprogramming by resolving cytokines (IL-4, IL-10). Front Cell Neurosci..

[46] Yang M-S, Park EJ, Sohn S (2002). Interleukin-13 and -4 induce death of activated microglia. Glia..

[47] Shin WH, Lee D-Y, Park KW (2004). Microglia expressing interleukin-13 undergo cell death and contribute to neuronal survival in vivo. Glia..

[48] Bal-Price A, Matthias A, Brown GC (2002). Stimulation of the NADPH oxidase in activated rat microglia removes nitric oxide but induces peroxynitrite production. J Neurochem..

[49] Ooboshi H, Ibayashi S, Shichita T (2005). Postischemic gene transfer of interleukin-10 protects against both focal and global brain ischemia. Circulation..

[50] Zhao X, Wang H, Sun G, Zhang J, Edwards NJ, Aronowski J (2015). Neuronal interleukin-4 as a modulator of microglial pathways and ischemic brain damage. J Neurosci..

[51] Liu X, Liu J, Zhao S (2016). Interleukin-4 is essential for microglia/macrophage M2 polarization and long-term recovery after cerebral ischemia. Stroke..

[52] Korhonen P, Kanninen KM, Lehtonen Š (2015). Immunomodulation by interleukin-33 is protective in stroke through modulation of inflammation. Brain Behav Immun..

[53] Kamel H, Iadecola C (2012). Brain-immune interactions and ischemic stroke: clinical implications. Arch Neurol..

[54] Zhang SR, Piepke M, Chu HX, et al. IL-33 modulates inflammatory brain injury but exacerbates systemic immunosuppression following ischemic stroke. JCI Insight. 2018; 3. 10.1172/jci.insight.121560 PMC6237219 30232272

